# Structure and State of Water in Branched *N*-Vinylpyrrolidone Copolymers as Carriers of a Hydrophilic Biologically Active Compound

**DOI:** 10.3390/molecules25246015

**Published:** 2020-12-18

**Authors:** Svetlana V. Kurmaz, Natalia V. Fadeeva, Vladislav M. Ignat’ev, Vladimir A. Kurmaz, Sergei A. Kurochkin, Nina S. Emel’yanova

**Affiliations:** 1 Institute of Problems of Chemical Physics, Russian Academy of Sciences, Prosp. Akad. Semenova 1, 142432 Chernogolovka, Russia; natali-vi@inbox.ru (N.V.F.); ignvm@74.ru (V.M.I.); kurmaz@icp.ac.ru (V.A.K.); oligo@icp.ac.ru (S.A.K.); n_emel@mail.ru (N.S.E.); 2 Department of Fundamental Physical and Chemical Engineering, M.V. Lomonosov Moscow State University, Leninskie Gory 1, 119991 Moscow, Russia; 3 Faculty of Fundamental Sciences, Bauman Moscow State Technical University, Baumanskaya 2nd 5, 105005 Moscow, Russia

**Keywords:** drug delivery, *N*-vinylpyrrolidone, amphiphilic copolymers, branching, hydrogen bond, quantum chemical modeling, metformin

## Abstract

Hydrated copolymers of *N*-vinylpyrrolidone (VP) with triethylene glycol dimethacrylate as a promising platform for biologically active compounds (BAC) were investigated by different physical chemical methods (dynamic light scattering, infrared spectroscopy, thermal gravimetric analysis, and differential scanning calorimetry) and the quantum chemical modeling of water coordination by the copolymers in a solution. According to the quantum chemical simulation, one to two water molecules can coordinate on one O-atom of the lactam ring of VP units in the copolymer. Besides the usual terminal coordination, the water molecule can form bridges to bind two adjacent C=O groups of the lactam rings of VP units. In addition to the first hydration shell, the formation of a second one is also possible due to the chain addition of water molecules, and its structure depends on a mutual orientation of C=O groups. We showed that *N*,*N*-dimethylbiguanidine hydrochloride (metformin) as a frontline drug for the treatment of type 2 diabetes mellitus can be associated in aqueous solutions with free and hydrated C=O groups of the lactam rings of VP units in studied copolymers. Based on the characteristics of the H-bonds, we believe that the level of the copolymer hydration does not affect the behavior and biological activity of this drug, but the binding of metformin with the amphiphilic copolymer will delight in the penetration of a hydrophilic drug across a cell membrane to increase its bioavailability.

## 1. Introduction

The most important criteria of polymer use in biomedical applications, partially as drug delivery [1,2,3,4,5,6], are their biocompatibility, as well as the ability to absorb them and form complexes of different kinds. Water adsorption precedes the adsorption of a synthetic polymer with proteins when it reaches a living organism, and the presence and state of water on a polymer surface may affect or even determine its biocompatibility [7,8,9,10,11,12,13,14,15,16]. One believes that hydrated polymers are able to participate in reactions of a complex formation with drugs via the associated water [17]. In this regard, great attention is paid to the study of the water structure in polymers used in biology and medicine [17,18,19,20,21,22,23,24,25,26,27].

In spite of a wide biomedical application of poly-*N*-vinylpyrrolidone (PVP) and its copolymers [28,29,30,31,32,33,34,35,36], the water structure and its state in them were studied insufficiently, in contrast to, e.g., polyethylene glycol (PEG) [37,38]. Meanwhile, investigations of PVP–water systems allowed to detect H-complexes that feature different stoichiometry and energy [17]. In normal conditions, the maximum PVP hydration degree consists of two water molecules per one polymer unit with respect to a cluster of four water molecules localized between two adjacent polymer units [17]. Water interactions with a polymer in solutions or at temperatures higher than its glass transition leads to a change of macromolecules conformation, i.e., the oncoming of pyrrolidone rings and straightening of the main chain. In this case, the water molecules bind to the polymer matrix rather strongly, up to the charge transfer. Water adsorbed at temperatures below the glass transition, which means conditions of invariable conformation of the polymer chains, bonded with a polymer matrix sufficiently weaker. The comparative study [39], with using IR spectroscopy and density functional theory calculations, demonstrated interactions between water molecules and PVP or its copolymers. Three types of water were found in a process of its adsorption by PVP. The absorption of water II appeared within the region 3300–3500 cm^−1^ and then type I water (vibrations below 3300 cm^−1^) and, after, the free or weakly bonded water III type (3500–3800 cm^−1^). According to the author’s opinion [28], VP (co)polymer biocompatibility is defined by strongly bonded water molecules.

In contrast to linear, amphiphilic VP copolymers of branched structures became a subject of detailed study due to the development of new, synthetic approaches; for instance, “Strathclyde methodology” [40]. It is one of the most simple and efficient methods to prepare branched copolymers that allows to control monomer composition, molecular weight and branching using commercial vinyl monomers. The radical copolymerization of ordinary vinyl(idene) monomers under conditions of the chain transfer allows the creation of the branching points using commercial multifunctional monomers as branching agents. The broad possibilities of macromolecular design allow to produce water-soluble branched copolymers sensitive to the temperature and medium pH changes [41,42,43].

We synthesized amphiphilic copolymers of VP with dimethacrylates [44] that were excellent precursors to form biologically active compound (BAC) nanostructures [45,46,47,48]. They have low cytotoxicity and are able to penetrate into cells [46]. For instance, they proved to be useful as potential carriers of hydrophobic compounds such as fullerene С_60_ [45] and zinc(II) tetraphenylporphyrin [46,47], and they were also approved as NO-carried systems of binuclear tetranitrosyl iron complexes [48] or Pt^IV^ complexes [49,50] with antitumor activity. Apparently, these copolymers could be also used as platform for hydrophilic BAC-forming complexes via associated water. For this reason, the experimental and theoretical study of water states in amphiphilic VP copolymers of branched structures present their current importance.

The goal of this study was to investigate the water structure and state in copolymers of VP with triethylene glycol dimethacrylate (TEGDM) using a set of physical chemical methods to carry out a quantum chemical analysis of H-complexes formed between amphiphilic copolymers and water molecules and to estimate the possibility of known antidiabetic drug *N*,*N*-dimethylbiguanidine hydrochloride (metformin, MET) binding with copolymers via hydrated water molecules.

## 2. Results and Discussion

### 2.1. Synthesis of VP-TEGDM Copolymers and Their Characterizations

**Scheme 1:**
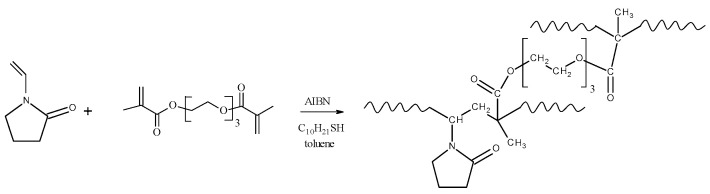
The synthesis of *N*-vinylpyrrolidone-triethylene glycol dimethacrylate (VP-TEGDM) copolymers via radical copolymerization under chain transfer conditions.

VP-TEGDM copolymers were obtained by radical copolymerization initiated by azobisisobutyronitrile (AIBN) in the presence of 1-decanethiol (DT) as a chain transfer agent to restrict the growth of the polymer chains, according to Scheme 1.

Under the specified conditions of synthesis, the copolymers completely soluble in toluene were obtained after copolymerization. In this case, no macrogels, i.e., insoluble products, were formed. From the reaction mixture, the copolymers were isolated by precipitation with hexane. The resulting copolymer fractions (soluble and insoluble in hexane) differed in molecular weight and composition. The results of the analysis of only high-molecular fractions (insoluble in hexane) are discussed in this article. The soluble-in-hexane fractions had a low molecular weight of about 1 kDa and high dimethacrylate unit contents, because they were not of interest for this study.

**Scheme 2:**
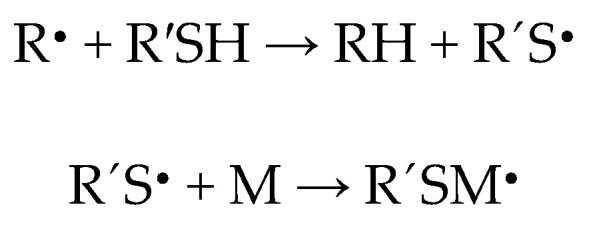
The polymer chain termination under the chain transfer reaction.

A 3D copolymer structure was formed after the polymerization of dimethacrylate as more active comonomer [44]. It contained pendant double bonds that mainly attached PVP chains. The polymer chain termination occurred under the chain transfer reaction (Scheme 2):

The first stage consists of an H-atom breaking from the DT molecule by growing radical R^•^ and leads to its deactivation. In the second stage, the radical C_10_H_21_S^•^ gives rise to a new kinetic chain. As a result, the material chain is limited, and the copolymerization rate decreases [44]. It results in producing a 3D structure with low-polar methacrylate nucleus and a polar shell formed by polymer chains consisting of VP fragments.

Amphiphilic copolymers are soluble in alcohols, water, chloroform, methylpyrrolidone, DMSO and MeCN. The contents of the С, Н, N and S atoms in copolymer **1** were 59.9%, 9.1%, 8.2% and 1.7%, respectively, and, in copolymer **2,** were found to be 59.9%, 9.2%, 8.2% and 1.7%, respectively. Their molar compositions were calculated from an elemental analysis. It showed that the contents of the VP and TEGDM units in copolymers **1** and **2** were 80.5 and 12.4 mol%, respectively. The DT residue (С_10_H_21_S groups) on the ends of the polymer chains was 7.1 mol%.

**Figure 1:**
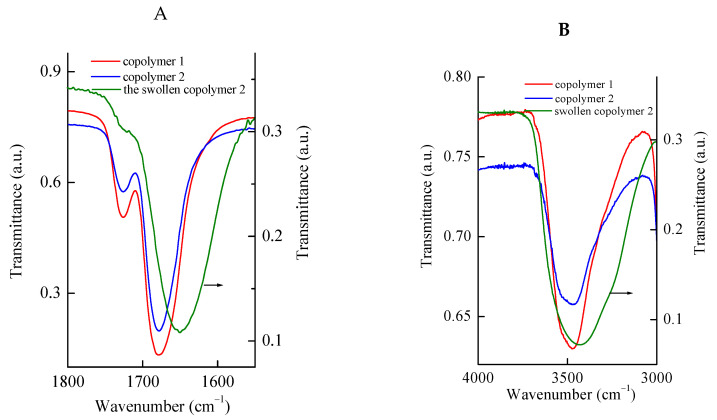
(**A**) IR spectra of the copolymers **1** and **2** films cast from chloroform and swollen in water copolymer **2** in the regions of 1600–1800 and (**B**) 3000–4000 cm^−1^.

IR and ^1^Н-NMR spectroscopy confirmed the copolymers composition. We observed absorption bands typical for C=O stretching vibrations in the methacrylate and VP units, respectively, at ν ~1725 and ~1680 cm^–1^ in the region of 1600–1800 cm^–1^ of the IR spectra (Figure 1). Signals related to monomer units were also observed in the ^1^Н-NMR spectra (Figure S1). Thus, there were two groups of bands related to VP units; the first set included signals due to NCH_α_ protons in the polymer chain and –CH_2_C=O fragments of pyrrolidone at *δ* = 3.0–4.0 ppm. The second one consisted of signals attributed to protons of CH_2_ in the polymer chain, as well as C–CH_2_–C and NCH_2_ fragments of pyrrolidone at *δ* = 1–3 ppm. Proton signals of the CH_3_ group in dimethacrylate units were detected in the spectrum of the copolymers at *δ* ~1 ppm. Signals at *δ* = 1.3 and 2.5 ppm might correspond to protons of (CH_2_)_8_ and CH_2_–S fragments of DT. Signals of hydrogen atoms in –CH_2_–CH_2_ fragments of dimethacrylate involved in the reaction were observed in ^1^H-NMR spectra at *δ* ~4.1 ppm. Some differences in the ^1^H-NMR spectra of copolymers could be associated with a topology of their macromolecules, e.g., with the presence of nanogel particles in copolymer **2**.

The absolute average molecular weight *M*_w_ of the copolymers and their polydispersity (PD) determined from the data of a two-detector size exclusion chromatography (SEC) are represented in Table 1.

Copolymers **1** and **2** are characterized by low molecular weights and high PD indices. The analysis of the molecular weight distribution curve (MWD) and weight average molecular weight *M_w_* at the eluent volume *V_R_* indicate a branched structure of their macromolecules. Figure S2A shows the MWD curves of the VP-TEGDM copolymers and linear PVP (*M_n_* = 9 кDа, PD = 4.6 (Refractive Index Detector, RI) and *M_n_* = 17 кDа, PD = 2.6 (RI + Multiangle laser light scattering, MALLS)). The MWD curve of the homopolymer PVP is unimodal in comparison with the copolymers. The MWD curves of the copolymer are a superposition of poorly resolved peaks of macromolecules of different compositions with similar molecular weights. The dependences of the molecular mass *M*_w_ on the eluent volume *V_R_* (Figure S2B) allow us to draw some conclusions about the copolymer topological structure. It can be seen that, in the range from 6.3 to 7.7 mL at the same *V_R_* value, macromolecule **1** elutes with a higher molecular weight and, consequently, a higher molecular packing density of the polymer chains vs. linear PVP elute. The Zimm factor *g*′ of copolymer **1** calculated for macromolecules with *M_w_* ~10^4^ as the ratio of its root mean square radius of gyration and linear PVP is 0.6–0.7 and definitely indicates branched character. Meanwhile, the dependence *M*_w_ (*V*_R_) of copolymer **2** lies lower than that of linear PVP, apparently due to cyclic structures of nanogels or microgels. It is no accident that the absolute molecular weight *M*_w_ (Table 1) of this copolymer is lower than that of copolymer **1**. In general, as follows from the analysis of the dependencies presented in Figure S2, the VP-TEGDM copolymers are a set of macromolecules with different molecular weights and topological structures. The copolymers were obtained under the same molar ratio of reagents but with different solvent concentrations. This allows us to conclude that the solvent concentration affects the macromolecules topology. From the data obtained, it follows that copolymer 2 with a lower molecular weight is formed at copolymerization in more concentrated solutions due to intramolecular cyclization and, apparently, contains more nanogel particles.

The copolymers are characterized with a low glass transition temperature Т*_g_* that is apparently caused by a large amount of end chains (Table 1). According to our data, the linear PVP of *M*_n_ ~(9−13) × 10^3^ and crosslinked poly-TEGDM have Т*_g_* from 125 to 150 °С and 113 °С, respectively.

**Figure 2:**
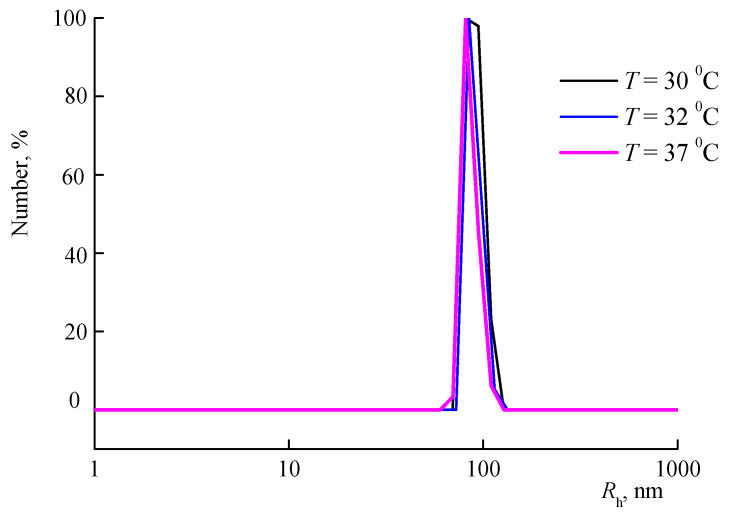
DLS curve of copolymer **1** in the aqueous solution at various temperatures.

We studied VP-TEGDM copolymer behaviors in polar solvents such as isopropyl alcohol and water by the DLS method. The amphiphilic VP-TEGDM copolymers exist in isopropyl alcohol (10 mg mL^−1^) as individual macromolecules of 4 to 5 nm size (Table 1)—namely, monomolecular micelles with shells formed by polar VP chains fragments and low-polar methacrylic cores. Aggregation processes are enhanced in water as a thermodynamically poor solvent for VP-TEGDM copolymers and aggregates with *R_h_* ~100 nm, i.e., multimolecular micelles [51], stabilized by hydrogen bonding in an aqueous solution of the copolymer (0.1 mg mL^−1^) existing at a temperature range of 20–37 °C (Figure 2). As the temperature increases to 40 °C, the size distribution of the polymer particles becomes bimodal, apparently due to the destruction of the aggregates. Particles with sizes of ~40 and ~200 nm appear in the water. Thus, the polymer particles are thermosensitive [41], and their dehydration is observed with increasing temperatures as a destruction of the hydrogen bond between the donor groups of the copolymer and the solvent molecules. The polar shell collapses, and the polymer particles aggregate.

**Figure 3:**
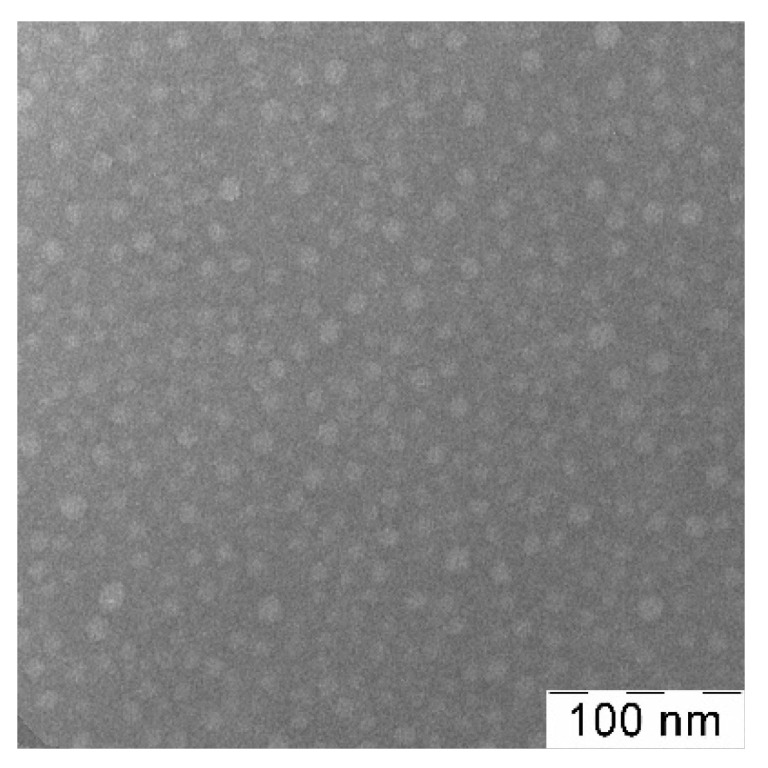
TEM image of copolymer **1** from the aqueous solution.

Of course, the dehydration of the copolymer takes place when the sample is dried before transmission electron microscopy (TEM) registration. Consequently, spherical particles with a diameter ca. 10–12 nm are visible in the image (Figure 3). The results obtained indicate that water affects significantly the behavior of the amphiphilic VP-TEGDM copolymer in the solution—namely, its ability to form aggregates and their stability.

Copolymers **1** and **2** keep high hydrophilicity and contain adsorbed water in a solid state. To determine the water content and its state in copolymers **1** and **2**, they were studied by a set of physical–chemical methods below and above the temperature of the glass transition. The samples were kept at normal conditions, i.e., temperatures of 20–25 °С and relative humidity of 50–70%.

In the IR spectra (Figure 1B, curves 1 and 2), films of the copolymers **1** and **2** dried at 60 °С showed a broad absorbance band in a 3400−3600-cm^−1^ area related to the stretching vibration of –ОН groups of adsorbed water. This band represents a superposition of several ones with maximums at ~3550, ~3460 and ~3340 cm^−1^. Apparently, they all should be attributed to the water associated with a copolymer via H-bonds of different strengths and energy of ~5–8 kcal mol^−1^ based on the wave numbers of the ОН-group vibrations [17]. A weak absorbance at 3670 cm^−1^ can be isolated in the IR spectrum of copolymer **1** specific for a free water (water III) С=О group of TEGDM. O-atoms in the –(СН_2_-СН_2_-О-)_3_ bridge between methacrylate groups, as well as С=О of VP unites, can evidently be the binding centers with water molecules in copolymers. The shift of frequency of a respective absorbance band of copolymer **2** film saturated with water vapors (Figure 1A, curve 3) from 1678 cm^–1^ to 1650 cm^–1^ indicates water binding with С=О of the lactam cycle. Moreover, the optical density of the C=O bond absorption band in TEGDM decreases, and the absorption band of the stretching vibrations of OH groups in copolymers also shifts to lower frequencies as a result of the formation of complexes with strongly bound water (water I and II).

**Figure 4:**
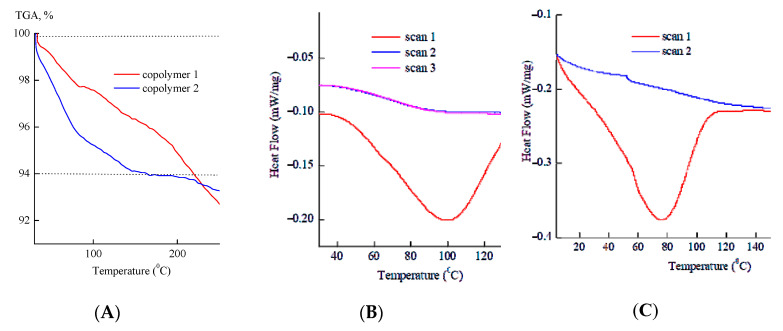
(**A**) Curves of the TGA and (**B**,**C**) DSC of copolymers **1** and **2.**

The TGA and DSC studies within a broad temperature range confirm the presence of water in the studied polymers. TGA curves of copolymers (Figure 4A) have a complicated nature. Within the temperature range of 40−150 °С, we can isolate some parts that apparently were caused by the removal of water. Molecular ions with *m*/*e* = 17 and *m*/*e* = 18 in their mass spectra support this claim.

The weight loss of copolymer **2** consists of ~6% with respect to ~0.74 water molecules per two VP units. Note that the equilibrium water content in PVP depends on conditions of its preparation and storage [17]. Depending on the air humidity, it can reach from 10 to 20 wt% in low-dispersed powder, i.e., from 0.76 to 1.54 water molecules that are fitted per one VP unit. The water content consists of 5–7 wt% or ca. 0.5 water molecules per one polymer unit in granulated PVP. Such PVP loses its mass up until 200 °С; on that account, a fast process is started already at 40 °С and finished at 100 °С and a slow one at 120–130 and 170–175 °С [17].

Taking into account the TGA data on the water content in the copolymers studied, we performed triple-scanning of copolymer **1** by DSC, taking into account the fact that PVP is considered as “dry” after the first scan of the sample up to 200° [17]. Moreover, the maximum of the endothermic peak on PVP thermograms is not a constant value and can be shifted to a higher temperature region, and weak endothermic peaks exist within the 120–130 and 160–175 °С regions. As it should be expected, a broad endothermic peak is observed under the first scanning of the copolymers on a DSC curve that is associated with the removal of water (Figure 4B,C). Poorly expressed steps on the thermograms in the low-temperature region under the second and third scanning were attributed to glass transition caused by defrosting of the polymer chain mobility. It shows, like in PVP, branched VP-TEGDM copolymers are getting “dry” after the first scanning. The equilibrium water content in copolymer **2** calculated from the integral value of this peak, taking into account the enthalpy of the evaporation of water (2.26 kJ g^−1^) [17], was ~4%. The maxima of the endothermic peak of the copolymers differed significantly and corresponded to ~100 and 75 °C, probably due to their different packing densities. Thus, VP-TEGDM copolymers form H-complexes of different energies with water, and as a result, the water is removed in a broad temperature range. This fact corresponds to the experimental data on PVP [17]; three states of water are distinguished in its first hydrate layer.

### 2.2. Quantum Chemical Modeling of H-Complexes of VP-TEGDM Copolymers

**Figure 5:**
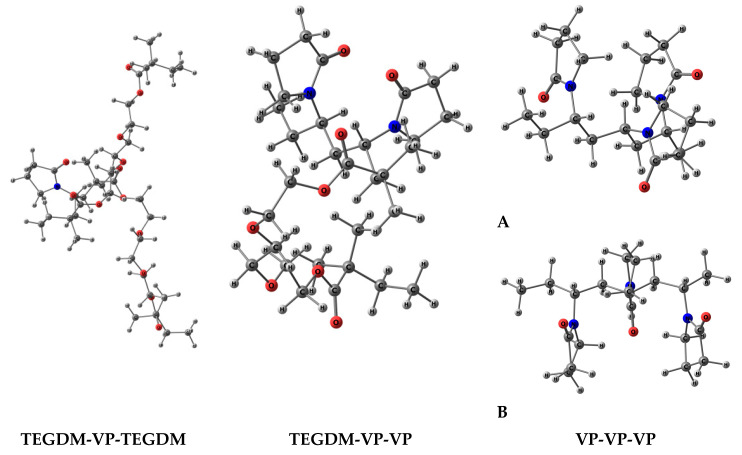
Optimized geometries of considered statistically possible sequences of the *N*-vinylpyrrolidone-triethylene glycol dimethacrylate (VP-TEGDM) copolymer.

The quantum chemical modeling was performed for various statistically possible addition sequences of monomeric unites of active TEGDM and VP monomers in a polymer chain based on their reactivity. The sequences were as follows: TEGDM-VP-TEGDM, TEGDM-VP-VP and VP-VP-VP. Their optimized geometries are represented in Figure 5. We did not consider other statistically feasible sequences, such as TEGDM-TEGDM-TEGDM, TEGDM-TEGDM-VP or TEGDM-VP-TEGDM. The discussion of all possible conformers existing in systems will be the subject of a new study. Therefore, we limited ourselves to the consideration of only one case—geometric isomers of the VP-VP-VP site. We analyzed “*trans*” and “*cis*” conformations when C=O groups of pyrrolidone rings were turned either into different (Figure 5A) or the same sides (Figure 5B). According to our calculations, the “*cis*” conformer is only 3.2 kcal mol^−1^ lower in energy; therefore, the hydrate complexes based on both conformers of the VP-VP-VP site were considered. The modeling of the structure of possible H-complexes was performed in a solvent media (water) within the framework of the polarizable continuum method (PCM).

**Figure 6:**
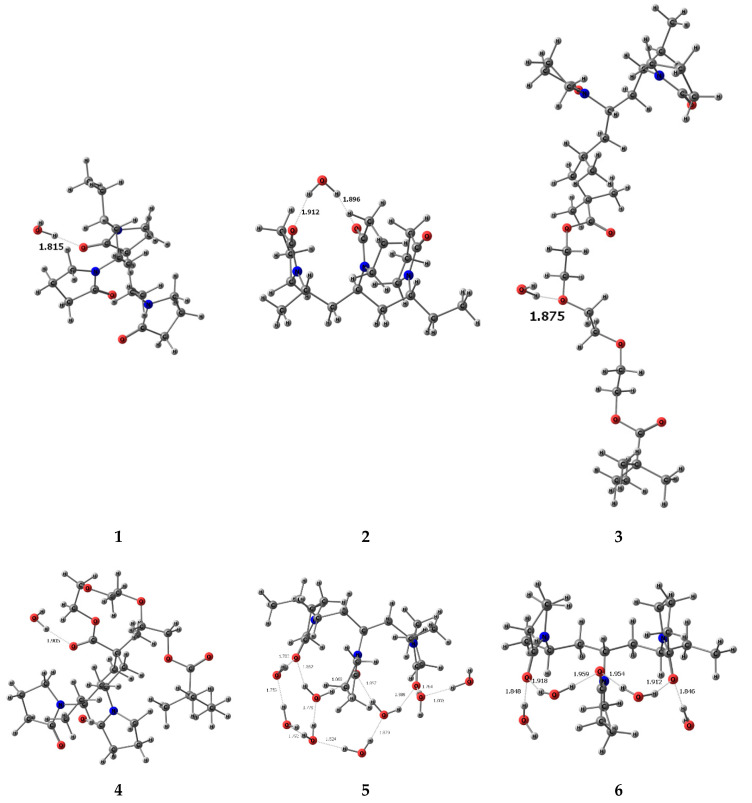
Optimized geometry of the VP-TEGDM sequences in the copolymer with water.

The optimization of the geometry was further performed for the most evident structures with water molecules. Some of them are represented in Figure 6. First of all, we considered the possibilities of individual functional groups of a copolymer interacting with water molecules. According to the calculations, water is able to form a hydrogen bond with the carbonyl oxygen atom of the lactam ring (structures **1** and **2**), the ether one (**3**) and carbonyl (**4**) oxygen atoms of TEGDM, as well as among themselves, forming the second hydrate shell (**5**). All these hydrogen bonds can be terminal (**1**), as well as bridging ones (**2**). The maximum of two water molecules are able to be coordinated at one O-atom of a functional group (**5** and **6**). The water molecules being immediately connected with a molecule of a dissolved compound form the first hydrate shell [17].

The water addition to the moiety of a copolymer forming hydrogen bonds is an exothermic process for all water molecule coordination. The addition enthalpy (−∆*H*) of one water molecule on the С=О groups of VP units for the VP-VP-VP copolymer moiety is 4−6 kcal mol^−1^ and 2−4 kcal mol^−1^ on the ether and carbonyl groups of the TEGDM unit. Insufficient differences in −∆*H* values did not allow unambiguous conclusions on the water molecule coordination. However, it was stated experimentally that these copolymers were monomolecular micelles, or their aggregates in aqueous solutions with hydrophilic shells consisted of VP unites [45,46,47,48]. Taking this data into consideration, the quantum chemical modeling of VP-VP-VP moieties with water molecules seems to be the most relevant.

Structures **1** and **2** (Figure 6) represent two types of additions of the first water molecule to the observed moiety. Bridge bonds between two C=O groups of the lactam ring are formed in the case of the *cis*-conformation of VP unites, when all oxygen atoms are turned onto the same side. The thermodynamic and energetic parameters of the process are given in the Table 2. It implies that formation of the bridge bond is more energetically favorable than a terminal one. The energy of one terminal bond is 9.0 kcal mol^−1^ from the quantum theory of atoms in molecules (QTAIM) calculations, while the energies of two hydrogen bonds forming a bridge bond in total are 15.9 kcal mol^−1^.

**Figure 7:**
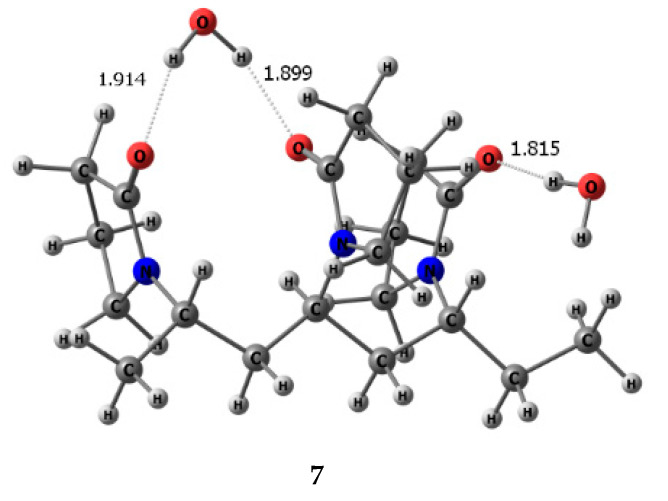
Optimized geometry of the VP-VP-VP copolymer moiety with 2 water molecules.

The addition of two water molecules (i.e., the terminal and a bridge one) to the VP-VP-VP moiety (Figure 7) is accompanied by release of 10.5 kсal mol^−1^. Lengths and energies of the hydrogen bonds do not change compared to the ones found for each coordination type in structures **1** and **2**. It is associated with the coordination of water via different C=O groups in this structure.

**Figure 8:**
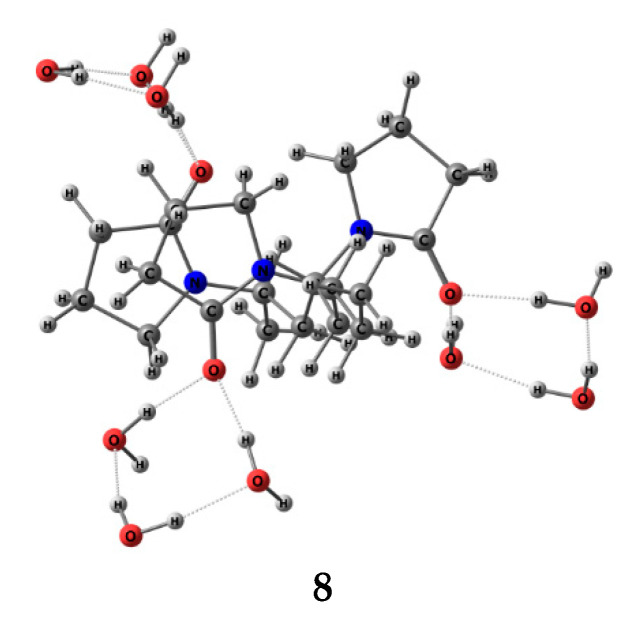
Optimized geometry of the VP-VP-VP∙9H_2_O in structure **8** with the *trans*-conformation of VP units.

In the case of the addition of three and four water molecules (structure **6**, Figure 6), the coordination of two water molecules at once on the oxygen of the С=О group of the lactam ring occurs by different means, as well as an increase of the −∆*H* value until 15.9 and 20.0 kcal mol^−1^, respectively. Evidently, H-bonds will be somewhat weaker, as follows from the values of their lengths (see structure **6**, Figure 6), i.e., at the coordination of two water molecules on one O-atom of the lactam ring, H-bonds became weaker with respect to the coordination on different O-atoms. Three water molecules did not coordinate with one oxygen atom, and our attempt to optimize such geometry led to the appearance of the second hydrate layer (Figure 8). Under this definition, we assumed such a coordination type where water molecules form hydrogen bonds with the hydration shell but not the polymer itself. This occurs even in the case when all coordination places are already occupied with water molecules. Since two water molecules to each C=O group in structure **6** were already added, hereafter, the chain addition of the water molecules is realized like in structure **5**. It is interesting to point out that the formation of the second hydrate shell leads to a bond strengthening in the first shell. As to the energetics of this reaction, the formation of the second hydrate shell is also an exothermic process. The value of −∆*H* was calculated for the addition of one water molecule to the first hydrate shell of the VP-VP-VP moiety, and it was demonstrated that the addition of the molecule to the terminal water liberated 4.6 kcal mol^−1^ and 5.3 kcal mol^−1^ in the case of the bridge one. As the result, water molecules form a cluster on the surface of this part of the polymer, and hydrogen bonds are formed between them as well.

Thus, water is localized in the copolymer in several ways due to the formation of hydrogen bonds between the hydrogen atom of the water molecule and the oxygen atom of the lactam ring or the oxygen atom of another water molecule. In the first case, the first hydration shell is formed. In the second case, it becomes possible to form a second hydration shell. Therefore, there is a water structuration under a polymer influence. It is a very important fact, because hydrophilic “guest” molecules can be easily coordinated on a surface of such a water “coat”. However, the hydration process of a С=О group of the lactam cycle of VP chains can deactivate these functional groups instead (Figure 8, structure **8**). In this case, “guest” molecules would be coordinated already on functional groups of TEGDM [52]. It should be pointed out that the formation of bridge bonds between C=O groups of the lactam ring is impossible in the case of *trans*-conformation of the VP-VP-VP moiety (Figure 8, structure **8**); according to our calculation data, its energy is only 3.2 kcal mol^−1^ higher than that of the *cis*-conformation. In this case, the second hydrate shell does not form a unified system and, at the same time, blocks the C=O groups.

**Figure 9:**
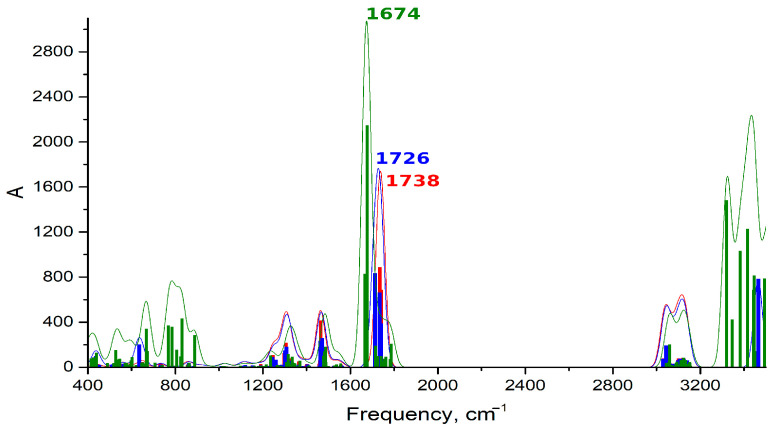
Simulated spectra of the VP-VP-VP moiety of the copolymer with *n* = 0, 1 and 8 water molecules respectively red, blue and green colors.

Simulated IR spectra of a copolymer segment of the VP-VP-VP type with water molecules are represented in Figure 9. It is observed that the addition of one water molecule to the С=О group of VP (structure **1,** Figure 6) creates bands in the simulated IR spectrum (it does not take into account a scaling multiplier) at 3350 cm^−1^ and 3654 cm^−1^ that are responsible for the stretching vibrations of O-H in the water molecule, and the weak one at 615 cm^−1^ is related to the hydrogen bond. The band of С=О vibrations of the VP fragment is shifted at 9 cm^−1^ into a low-frequency region. Its shift is already observed at 64 cm^−1^ with the addition of eight water molecules. Respectively, the band intensity considerably increases within the 3500 cm^−1^ region (Figure 9).

Large amounts of hydrogen bonds in structure **5** (Figure 6), containing eight water molecules, causes the appearance of sufficiently intensive bands within the 600−800 cm^−1^ region. Thus, the shift of the C=O group vibrations to a low-frequency region is increased with the number of increasing water molecules conjugated to the copolymer moiety. In the experimental IR spectrum of a copolymer isolated from water, the frequency of the stretching vibration of the С=О bond in the lactam cycle can reach ~1660 cm^−1^ [48]. In this case, conformation-sensitive bands within the skeleton vibration area (1100–800 cm^−1^) disappeared as a result of a change of a spiral conformation of “dry” PVP chains [17]. Apparently, the distance between rings was diminished as a binding result of two PVP chains by a water molecule that leads to the straightening of a polymer chain.

### 2.3. Quantum Chemical Modeling of MET-Copolymer Complexes

Based on quantum chemical modeling, some possible structures of MET complexes with a VP-TEGDMA copolymer in aqueous solutions were proposed [53]. Calculations show that MET can be coordinated via NH_2_ groups in a number of ways: mono- and bidentate; on VP (by the carbonyl group) and TEGDMA copolymer units (via either the ester or ether group); or in the case of bidentate coordination—due to one, neighboring or remote NH_2_ groups. The most energetically favorable and stable structure is one with the coordination of neighboring NH_2_ groups by the carbonyl oxygen of the VP fragment.

**Figure 10:**
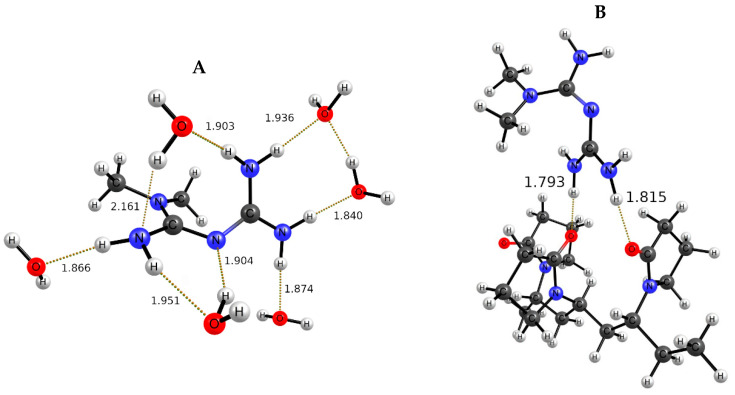

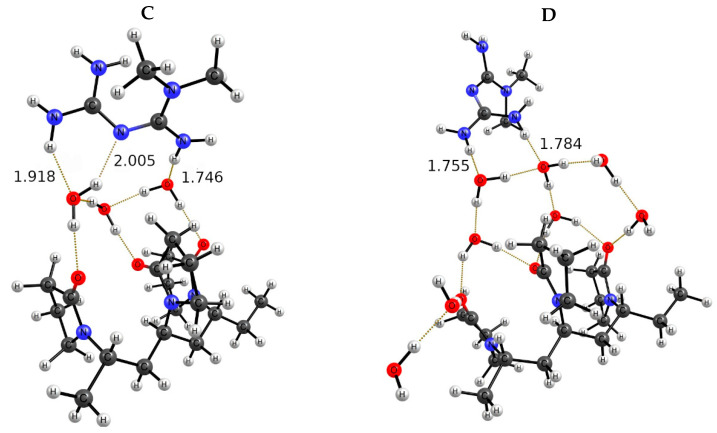
Optimized geometries of MET-copolymer structures: (**A**) MET with 6 water solvation molecules, (**B**) МЕТ associated with С=О groups of the lactam rings of the copolymer, (**C**) МЕТ associated with water molecules of its first hydration shell and (**D**) МЕТ associated with water molecules of its second hydration shell (the distances are given in Angstroms).

Here, we considered the MET bonds formed directly with the PVP moiety and its first and second hydration shells (Figure 10). Bonds of hydrated MET with water are shown for comparison. According to the quantum theory of atoms in molecules, all these bonds are typical hydrogen ones [54]. The arguments for it are as follows: the presence of critical bond points (3, −1), significantly lower values of the electron density (ρ) at the critical point in comparison with covalent bonds, weak positive values of the Laplacian of electron density (∇^2^ρ), mutual penetration of the basins of the hydrogen atom and the atom-acceptor of hydrogen bond, an increase in the charge of the hydrogen atom forming the bond.

The bond energy, like other parameters characterizing the bond strength (ρ and ∇^2^ρ) in the complexes of MET with water and complexes of MET with the PVP moiety, lie approximately in the same range, from 7.7 to 9.9 kcal/mol for the bonds of MET with water and from 9.4 to 9.9 kcal/mol for the bonds of MET with the PVP site. Hydrogen bonds in the complexes of MET with the first and second hydration shells of the PVP moiety are within the energy range from 7.7 to 11.6 kcal/mol and from 10.6 to 11.8 kcal/mol, respectively, which makes the formation of these bonds slightly more energetically favorable. This phenomenon may be explained by the main contribution to the increase in the hydrogen bond energy in chains, and clusters are created by a charge transfer along the chain, which leads to an increase in both the basicity of the lone electron pair and the acidity of the proton participating in the hydrogen bond [55,56]. However, these results do not allow us to conclude that the structure of these complexes is more stable than that of MET with a PVP fragment or water.

Since all characteristics of H-bonds are kept regardless of whether the drug is bound directly to the polymer or its first/second hydration shell, we believe that the level of the copolymer hydration does not affect the behavior and biological activity of the drug. The values of the calculated energies of the hydrogen bond indicate its equilibrium character; therefore, the equilibrium shifts towards free MET with the temperature increase. The theoretical assumptions were confirmed by experimental data [57]. We studied the complexes of metformin and the VP-TEGDM copolymer. The MET nanostructures of a small size (*R*_h_ ca. 50 nm) were prepared in a dilute solution of *N*-vinylpyrrolidone with a triethylene glycol dimethacrylate copolymer. The MET molecules were adsorbed on the copolymer or encapsulated in their internal cavities and associated via hydrogen bonds with free and/or hydrated C=O groups of the lactam cycle of VP units. At the physiological conditions, MET was released from the biocompatible polymeric carrier and acted as free MET on the blood glucose level in streptozotocin-treated diabetic mice [57].

## 3. Materials and Methods 

### 3.1. The VP-TEGDM Copolymer Preparation

*N*-vinylpyrrolidone (“Alfa Aesar”, Heysham, UK) was distilled in vacuum to remove inhibitor NaOH. TEGDM (“Aldrich”, Sigma-Aldrich, Inc., St. Louis, MO, USA) and 1-decanethiol (DT, “Alfa Aesar”) were used as received. Two kinds of copolymers (copolymers **1** and **2**) were obtained by radical copolymerization at a molar ratio of (VP):(TEGDM):(DT) = 100:12:12 in the presence of 75 and 60 vol% toluene, respectively. After copolymerization, the high molecular weight fraction was isolated from the mixture by precipitation in a tenfold excess of hexane. The copolymers were dried to a constant weight under vacuum to remove toluene and hexane. The yield of copolymers **1** and **2** was ~80%.

### 3.2. Elemental Analysis

The contents of the C, H, N and S atoms in the copolymers were determined by elemental analysis on a CHNS/O instrument Vario microcube Elementar GmbH (Elementar Analysensysteme GmbH**,** Hanau, Germany).

### 3.3. Size-Exclusion Chromatography

The molecular weight of the copolymers and linear PVP was determined by SEC on a Waters GPCV 2000 chromatograph (2 PS gel columns, 5 μm, MIXED-C, 300 × 7.5 mm Waters, Milford, MA, USA), as described in [46] using two detectors (RI + MALLS)—refractometric and WYATT DAWN HELEOS II light scattering (λ = 658 nm), software Empower Pro and Astra (5.3.2.20 version). The molecular weights were measured at a temperature of 70 °C; LiCl (1% wt.) was added in *N*-methyl pyrrolidone as eluent to avoid the macromolecule aggregation. The flow rate was 1 mL/min. 

### 3.4. IR Spectroscopy and ^1^H-NMR Study

The copolymers were identified by Fourier-transform infrared spectroscopy (FTIR) on a Bruker α spectrophotometer in transmission mode and ^1^H-NMR on a superconducting pulsed broadband two-channel AVANCE III 500 MHz Bruker BioSpin NMR spectrometer. The concentration of copolymers in deuterated chloroform was 6 mg mL^−1^. For IR spectral analysis, copolymer films were prepared using chloroform on KBr glasses. The swollen in water copolymer was examined between the plates.

### 3.5. Thermogravimetric Analysis

The thermal properties of the copolymers were studied using a thermogravimetric analysis on a synchronous thermal analyzer STA 409C LUXX NETZSCH (NETZSCH, Selb, Germany) in argon with a stepwise temperature rise from 30 to 500 °C and a heating rate of 5 °C min^−1^. Sample weight was ~10 mg. 

### 3.6. Differential Scanning Calorimetry

The glass transition temperatures *T*_g_ of the copolymers were measured using the DSC method on a METTLER TOLEDO STARe (Mettler-Toledo International Inc, Columbus, OH, USA) instrument module DSC 822 at a heating rate of 5 degrees min^−1^. The *T*_g_ value was determined according to the DIN standard.

### 3.7. Dynamic Light Scattering

The dynamic light scattering method was used to determine the sizes of scattering centers in a branched copolymer solution (isopropyl alcohol and water) at the detection angle of 90°. We used a Photocor Compact (LTD Photocor, Moscow, Russia) equipped with a diode laser operating at a wavelength of 654 nm. The solutions of the copolymers were previously passed through a filter with pores of 0.45 μm in diameter. Before measuring, the vial with the solution was thermostated for ca. 20 min. The hydrodynamic radii *R*_h_ of the copolymers were calculated using the Einstein-Stokes Equation (1)

(1) D= k×T6×π×η×R 

where *D* is the diffusion coefficient, *k* is the Boltzmann constant, *T* is the absolute temperature and *η* is the viscosity of the medium in which the dispersed particles are suspended.

### 3.8. Transmission Electron Microscopy

TEM images of the VP-TEGDM copolymer from the aqueous solution (0.1 mg mL^−1^) were obtained by Leo 912 AB equipment (Leo Electron, Carl Zeiss AG, Hsu Koehn, Germany). Phosphotungstic acid was used to supply a contrast of the sample.

### 3.9. Simulation Protocols

By using the Gaussian 09 program (Version D) [58], quantum–chemical calculations were performed within the framework of the density functional theory (DFT), with the full optimization of the geometric parameters of the section of the polymer chain, consisting of three units of monomers. This was enough to model our systems and did not require significant computer resources. The second step was to put from one to nine molecules of water on this section of the polymer chain and to optimize the obtained structures. In the best functional that gives the most adequate description of the available experimental data, we searched for the minimum scaling factor when comparing the experimental and theoretical IR spectra. In this case, the counting rate was taken into account. It was found that the meta-hybrid TPSSh functional [59], in combination with the standard 6-311++G **//6-31G* basis set, turned out to be the most acceptable. The influence of water as a solvent was taken into account using the model of the polarizable continuum method (PCM). The results of the calculations showed no imaginary frequencies, and all the optimized structures corresponded to the minimum potential energy. To analyze the wave function by methods of the QTAIM, we used the AIMAll program package (Version 10.05.04) [60]. The wave functions of the structures were calculated in the same approximation as the optimization of the geometric parameters. The energies of the intermolecular bonds were calculated using the known correlation formula Espinosa-Lecomte [61] *E*_A-B_≈1/2*v*_e_(*r*), where *E*_A-B_ is the energy of the A-B bond and *v*_e_(*r*) is the potential energy density at the critical point of this bond. The processing of simulated IR spectra was completed using the ChemCraft program, version 1.8 [62].

## 4. Conclusions

The experimental data obtained by the IR spectroscopy, TGA and DSC and quantum chemical modeling results demonstrated the ability of amphiphilic copolymers of *N*-vinylpyrrolidone with triethylene glycol dimethacrylate to form H-complexes of different compositions and energies with water molecules. According to the calculations, the process of water coordination on a copolymer moiety containing three VP units is exothermic and occurs with the energy release. The coordination occurs on the oxygen of the С=O bond of the lactam ring. One to two water molecules can be coordinated on one oxygen atom. Besides the usual terminal coordination, a water molecule is able to form bridges bonding two adjacent C=O groups. In addition to this first hydrate shell, the formation of the second one is also possible through the chain addition of water molecules, although the bond energies are insufficiently weaker than in the first one. Its structure depends on a mutual orientation of the С=О groups. The observed effects in the TGA, DSC and DLS experiments were apparently caused by the destruction of the second hydrate shell as a result of the water break-off and destruction of the first hydrate shell at higher temperatures. According to quantum chemical modeling, metformin as a drug can be associated with copolymer functional groups or its hydrated water in aqueous solutions. All characteristics of H-bonds are kept regardless of whether the drug is bound directly to the polymer or its first/second hydration shell. This allows us to conclude that the level of copolymer hydration does not affect the behavior and biological activity of the drug, and, at physiological conditions, MET will be release from the biocompatible polymeric carrier and act as a free MET on the blood glucose level. The addition of metformin to an amphiphilic copolymer will delight in the penetration of any drug across a cell membrane to increase its bioavailability. Due to their smaller size and globular nature, amphiphilic VP copolymers represent significant interest as a modern platform for BAC molecules of different natures and, therefore, can be used in various biomedical applications.

## Figures and Tables

**Table 1:** Physicochemical characteristics of the N-vinylpyrrolidone-triethylene glycol dimethacrylate (VP-TEGDM) copolymers.

The Copolymers	*M_w_* × 10^−3^ g mol^−1^ (RI + MALLS *)	PD	R_h_ **, nm	Т*_g_*, °С
**1**	19.9	3.4	4.0	63
**2**	16.0	3.5	5.0	74

* The increment of the refractive index d*n*/d*c* was in the range of 0.056–0.058 mL g^−1^. ** Measurements were carried out in 1 wt% solutions of copolymers in isopropyl alcohol at a temperature of 30 °C. M_W_: molecular weight and PD: polydispersity.

**Table 2:** Thermodynamic and energy parameters of the attachment of one water molecule to the VP-VP-VP moiety. QTAIM: quantum theory of atoms in molecules and −∆*H*: addition enthalpy.

The Structure	A Type of Water Connection					
	Terminal Connection			Bridge Connection		
	−∆*H*, kcal mol^−1^	*Е(QTAIM)*, kcal mol^−1^	The Length of О…НBond, Å	−∆*H*, kcal mol^−1^	*Е(QTAIM)*, kcal mol^−1^	The Length of О…НBond, Å
**1**	4	9.0	1.815	-	-	-
**2**	-	-	-	6.5	8.1; 7.8	1.912; 1.896

## Supplementary Materials

The following are available online, Figure S1: ^1^Н-NMR spectra of copolymers **1** and **2** in deuterated chloroform; molecular mass distribution curves of copolymers **1** and **2** and PVP. Figure S2: Molecular mass distribution curves of copolymers **1** (*1*) and **2** (*2*) and PVP (*3*) (A) and their molecular mass *M_w_* at the eluent volume *V_R_* in the semilogarithmic coordinates (B). Table S1: Parameters of hydrogen bonds received from the QTAIM.
