# Supplementary material for: Structure and State of Water in Branched N-Vinylpyrrolidone Copolymers as Carriers of a Hydrophilic Biologically Active Compound
Source: Molecules. 2020 Dec 18;25(24):6015. doi: 10.3390/molecules25246015 (PMC7765915; doi:10.3390/molecules25246015)
Supplement: Supplementary file 1 [file molecules-25-06015-s001.pdf]

molecules

Supporting information

Supplementary data including ^{1}H NMR spectra of copolymers **1** and **2** in deuterated chloroform; molecular mass distribution curves of the copolymers **1**, **2** and PVP, their molecular mass M_w at eluent volume V_R in semilogarithmic coordinates, and the Table containing hydrogen bonds parameters are available online.

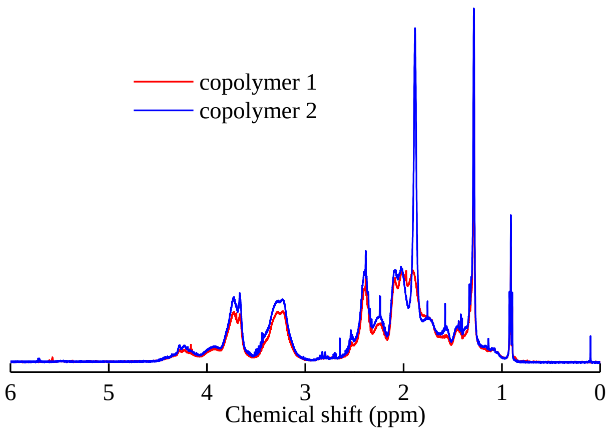

Figure S1. ^{1}H NMR spectra of copolymers **1** and **2** in deuterated chloroform.

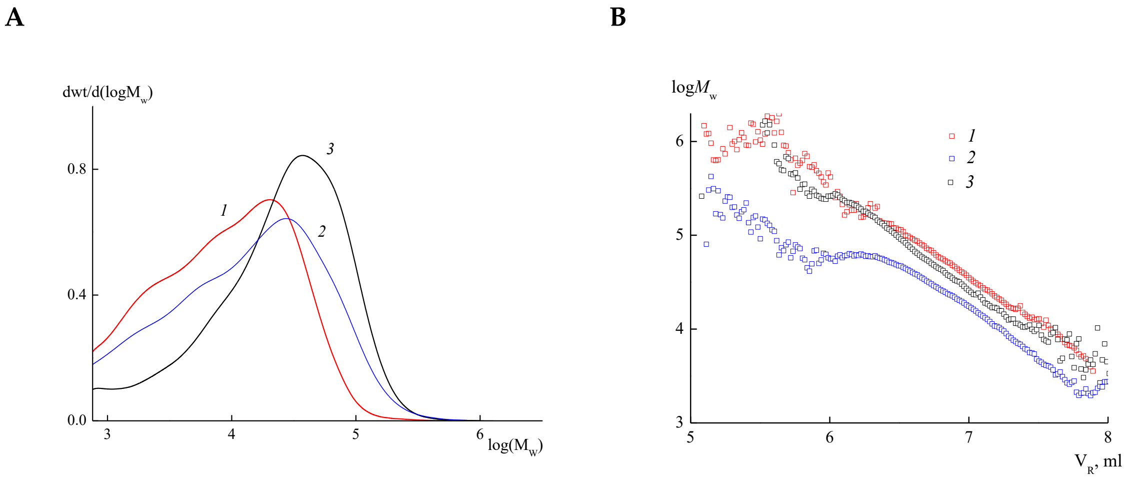

Figure S2. Molecular mass distribution curves of the copolymers **1** (*1*), **2** (*2*) and PVP (*3*) (**A**), their molecular mass M_w at eluent volume V_R in semilogarithmic coordinates (**B**).

Table S1 Parameters of hydrogen bonds received from QTAIM

Structures (Figure 10)	ϱ	∇²ϱ	E_{bond}, kcal/mol
a	0.037	+0,101	9.9
	0.034	+0,094	8.9
	0.034	+0,095	8,6*
	0.030	+0,082	7,8
	0.034	+0,098	9.0
	0.021	+0,058	4,8*
	0.032	+0,089	8,5
	0.028	+0,084	7,7
b	0,036	+0,109	9,4
	0,038	+0,114	9,9
c	0,045	+0,120	11,6
	0,027	+0,080	6,7*
	0,029	+0,090	7,7
d	0,040	+0,116	10,6
	0,045	+0,120	11,8

*The overwhelming majority of the bonds under consideration are formed by the hydrogen of the amino groups of metformin and the oxygen of water or oxygen of the lactam cycle of vinylpyrrolidone. The noted bonds are the exception. In those bonds not oxygen, but the nitrogen atom of metformin acts as the acceptor of the hydrogen bond. Those bonds stand out from the whole number of presented bonds. Lower values of the moduli of ϱ, ∇2ϱ and E_{bond}, which indicates a lower strength of those hydrogen bond [54]. Hydrogen bonds formed by nitrogen atoms, due to the lower bond strength and their relative rarity, make a significantly smaller contribution to the stabilization of intermolecular metformin complexes, therefore we do not take them into account.
